# Safety classification of herbal medicines used among pregnant women in Asian countries: a systematic review

**DOI:** 10.1186/s12906-017-1995-6

**Published:** 2017-11-14

**Authors:** Mansoor Ahmed, Jung Hye Hwang, Soojeung Choi, Dongwoon Han

**Affiliations:** 1 Department of Global Health and Development & Department of Preventive Medicine, Hanyang University, College of Medicine, 222 Wangsimni-ro, Seongdong-gu, Seoul, 04763 South Korea; 2 Institute of Health Services Management, Seoul, South Korea; 3 Department of Obstetrics and Gynecology, Hanyang University, Seoul, South Korea

**Keywords:** Pregnancy, Herbal medicines, Adverse effects, Safety, Asian countries

## Abstract

**Background:**

High prevalence of herbal medicines used in pregnancy and the lack of information on their safety is a public concern. Despite this, no significant research has been done regarding potential adverse effects of using herbal medicines during pregnancy, especially among developing Asian countries.

**Methods:**

Cross-sectional studies were searched up to year 2016 on PubMed/Medline and EMBASE, the data were extracted and quality of studies was assessed using the quality appraisal tool. The findings are reported in accordance to the PRISMA checklist (Preferred Reporting Items for Systematic Reviews and Meta-Analyses). Classification on safety of identified herbal medicines was done based on current scientific literature.

**Results:**

This study included eight cross-sectional studies (2729 participants) from seven different Asian countries, of which 1283 (47.01%) women used one or more herbal medicines during pregnancy. Peppermint (22.8%), aniseed (14.7%), olibanum (12.9%), flixweed seed (12.2%) and ginger (11.5%) were the most frequently used herbal medicines. Out of the 33 identified herbal medicines, 13 were classified as safe to use, five as use with caution, eight were potentially harmful to use in pregnancy and information on seven herbal medicines was not available in the current literature.

**Conclusions:**

Several herbal medicines identified in this review were classified to be potentially harmful or the information regarding safety in pregnancy was missing. It is recommended that contraindicated herbal medicines should be avoided and other herbals should be taken under supervision of a qualified health care practitioner. The classification regarding safety of herbal medicines in pregnancy can be utilized to create awareness on prevention of adverse effects.

**Electronic supplementary material:**

The online version of this article (10.1186/s12906-017-1995-6) contains supplementary material, which is available to authorized users.

## Background

Asian countries have a long history of using traditional herbal medicines to manage various medical conditions [1]. A multiethnic study reported that 50% of the Asians consumed one or more herbal products to manage their health [2]. In Asian countries, medicinal plants and their preparations can be easily purchased from condimental shops and homeopathic stores for self-treatment or can be obtained by visiting traditional healers [3]. Herbs can also be obtained as unregulated food products which usually do not go through standard regulatory process [4]. Consequently there is always a risk of contamination with heavy metals or undeclared pharmacological agents [5].

Despite such risks, herbal medicines are popular among pregnant women [4]. Its prevalence up to 60% in the developed countries [6] is mainly because of the belief that herbs are natural and free of any adverse effects compared to conventional medicine [7]. Local traditions and social pressure could also be the reason behind this practice [8].

These socio-cultural factors may affect the outcome of pregnancy [9–11]. For instance, one study showed that women using herbal medicines during pregnancy had higher incidence of threatening miscarriage, and newborns of herbal users were smaller for their gestational age [12]. The authors hypothesized that such association could be result of the regular intake of chamomile and licorice throughout the course of pregnancy. Other studies have also evaluated possible adverse effects of using various herbal medicines during pregnancy [13, 14]. Amid these safety concerns, few studies have classified the commonly used herbal medicines in pregnancy according to their safety status.

Nordeng et al. reported that 39% of pregnant women used herbal medicines that were potentially harmful or for which sufficient information on safety was unavailable [15]. More recently, Kennedy et al. classified the safety of most commonly used herbal medicines during pregnancy in American, Australian and European populations [16]. However, the herbal medicines used in Asian population should be different from those used in western countries due to difference in culture and traditions [17]. Several studies from Asian region have indicated use of herbal medicines during pregnancy [18, 19] but no attempt has been made to classify the safety of herbal medicines used in pregnancy among developing Asian countries. This study has two aims. The first is to identify the most commonly used herbal medicines in pregnancy among developing Asian countries through a systematic review. The second is to classify the identified herbal medicines according to their safety status.

## Methods

This systematic review conformed to the methodological guidelines of Cochrane Handbook for Systematic Review. The findings are reported in accordance to the PRISMA checklist (Preferred Reporting Items for Systematic Reviews and Meta-Analyses) [20]. The checklist is given in the Additional file 1. For this study, the question was posed: “What is the current scientific evidence regarding safety of commonly used herbal medicines during pregnancy in developing Asian countries?”

### Type of studies

To identify commonly used herbal medicines among target population, the population-based cross-sectional studies were reviewed. Cross-sectional studies were selected because such studies typically report a large variety of herbal medicines [21–23]. It was hypothesized that such studies would allow authors to identify and subsequently classify as many herbal medicines as possible. Moreover, cross-sectional studies are frequently conducted to study health behaviour among pregnant women. The rationale to restrict selection to cross-sectional studies was to make the findings of the reviewed studies comparable.

### Search strategy

MA and SJ performed independent search on PubMed/MEDLINE and EMBASE (Ovid) for articles published from 2000 to 2016. PubMed/MEDLINE search strategy is presented in Additional file 2 as Table S1. The search was conducted using Boolean operators and different keywords alone and in combinations were used, with special focus on developing Asian countries.

### Eligibility criteria

Original research in human pregnancy based on cross-sectional survey were considered eligible to be included in this review. Additional criteria were reporting of every herb’s name and number of users.

### Safety documentation of identified herbal medicines

To classify safety of identified herbal medicines, several reference sources were used in order to gain multiple perspectives. These sources were: Herbal Medicines in Pregnancy & Lactation [24], Botanical Safety Handbook [25], The European Medicines Agency [26], database of Natural Medicines [27], PDR for Herbal Medicines [28], and the previously published safety classification by Kennedy et al. [16]. For those herbal medicines which were not listed in the above mentioned sources, other references were reviewed: Natural Standard Herb & Supplement Reference: Evidence-based Clinical Reviews [29] and Herbal Drugs and Phytopharmaceuticals: A Handbook for Practice on a Scientific Basis [30]. In case of any discordant information in the reviewed sources, being the more recent work on safety classification of herbal medicines used in pregnancy – study by Kennedy et al. [16] was used as primary reference source followed by Herbal Medicines in Pregnancy & Lactation [24].

If any of the identified herbal medicines was not listed in the reference sources, PubMed/MEDLINE and EMBASE (Ovid) were searched using the search strategy presented in Additional file 2 as Table S2. The search was performed from inception to August 2017. While classifying the safety of herbal medicines, evidence from human studies was considered first, followed by animal studies. If an herbal medicine was composed of two or more herbs, each herb was individually evaluated and classified. Based on a recent study [16], the identified herbal medicines were classified into four groups. Description of how these classifications were defined is presented in Table 1.

**Table 1:** Safety classification of identified herbal medicines

Classification	Description
Safe to use in pregnancy	Available human evidence suggests the herb can be safely used in pregnancy.
Use with caution	Available human evidence for the herb is limited so it should not be used without consulting a qualified health care practitioner.
Potentially harmful to use in pregnancy	Available evidence has shown adverse impacts on pregnancy or fetus following the use of the herb
Information unavailable	No reference was found regarding use of the herb in pregnancy

It should be noted that an herbal medicine could be the individual herb or the mixture of several herbs. Although this study classified safety status of each herb, the term ‘herbal medicine(s)’ is used throughout the paper for the purpose of simplicity and uniformity.

### Data extraction and management

Using eligibility criteria, two reviewers (MA and SJ) extracted data and piloted key information on a review template that was developed for this research. The data extracted from each study were compared for results and any discrepancies found by the two reviewers were resolved by the senior researcher (DW).

## Results

### Selection of studies

**Fig. 1:**
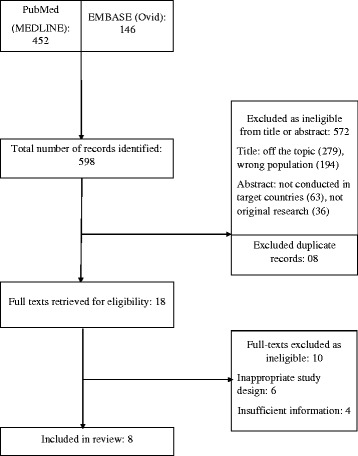
Systematic review flowchart

Flowchart of the studies included in this systematic review is illustrated in Fig. 1. The initial search of the databases yielded 598 records, of which eight records were duplicate and 572 others were excluded as ineligible after reading their titles or abstracts. Full texts of the remaining 18 records were downloaded and screened or in some cases, the full texts were screened online. After screening through eligibility criteria, 10 studies were considered ineligible. Therefore, eight studies were found eligible and were included in the systematic review.

### Study quality

To evaluate quality of the included studies, a quality assessment tool was used which was adapted from previous studies (Table 2) [31, 32]. The tool is used to indicate the methodological quality and appropriateness of the observational studies, including cross-sectional studies that were reviewed in this study. It consists of ten items, of which five items are standard and relate to general methodological aspects. Whereas the other items are modifiable so that reviewed studies’ relevance to the systematic review can be recognised. The score is presented as a percentage, where high score indicates higher quality of a study and vice versa. Two of the eight studies were moderate in terms of methodological quality whereas six others were strong. Studies with moderate score did not report response rate, the time of pregnancy when a certain herbal medicine was used or prevalence of each herbal medicine. The definition of herbal medicine in selected studies was comparable as it was according to the World Health Organization’s definition: “any medicinal product based on herbs, herbal materials, herbal preparations and finished herbal products, that contain as active ingredients parts of plants, other plant materials, or combinations thereof” [33].

**Table 2:** Quality Assessment of the Studies

No.	Study	Quality Assessment Items					Relevance to Current Study					% score^a^
		A	B	C	D	E	F	G	H	I	J	
1	Al-Riyami et al. [41]	1	1	1	1	1	1	1	0	0	0	70.0
2	Jaradat et al. [17]	1	1	1	1	1	1	1	1	1	0	90.0
3	Amasha et al. [18]	1	0	1	1	1	1	1	0	0	0	60.0
4	Hashem Dabaghian et al. [36]	1	1	1	1	1	1	1	0	0	1	80.0
5	Hwang et al. [38]	1	1	1	1	1	1	1	0	0	0	70.0
6	Orief et al. [49]	1	0	1	1	1	1	1	0	0	1	70.0
7	Rahman et al. [19]	1	0	0	1	1	1	0	1	0	0	50.0
8	Tabatabaee [42]	1	1	1	1	1	1	1	0	0	1	80.0

^a^Total score divided by the total number of items multiplied by 100

0—no or not reported; 1—yes

A—was sample likely to be representative of the study population? B—was the response rate mentioned in the study? C—was the instrument used reliable? D—was the ethical approval mentioned in the study? E—was it a primary data source? F—were names of used herbal medicine described? G—was prevalence of each herbal medicine used mentioned? H—was route of administration of each herbal medicine described? I—was outcome of pregnancy reported? J—was time of use of herb reported?

Quality assessment and relevance to the current study score: weak: 0–33.9%, moderate: 34%–66.9%, strong: 67%–100%

### Characteristics of studies

Out of the eight studies included in this review, two were conducted in Iran, and one each in Malaysia, Palestine, Iraq, Jordan, Oman and Egypt (Table 3). The study from Egypt was included due to country’s cultural proximity to the Arab countries included in this review. The study from Malaysia was the oldest in terms of year of publication (2009) and the most recent one was from Iraq (2016). All of the included studies used structured or semi-structured survey questionnaires to collect the data. One study from Iran employed the largest sample size of 600 women and it showed the highest (67%) prevalence of herbal medicine use, whereas the study from Oman recruited only 139 participants.

**Table 3:** Details of studies included in the review

(Author. Year)	Study objective	Study location	Study design; source of study subjects	Sample size (Mean age)	Herbal use N (%)
Al-Riyami et al. 2011 [41]	To evaluate medication use pattern in a university tertiary hospital in the Sultanate of Oman.	Oman	CSS; using structured questionnaire, women attending antenatal clinic	139 (28 ± 5)	33 (23.8%)
Jaradat et al. 2013 [17]	Aims of this study were to measure the prevalence and predictors of herb use among a group of Palestinian pregnant women and the possible influence of herbal consumption on pregnancy outcomes.	Palestine	CSS; using questionnaire, women in postnatal ward of a public hospital	300 (NR)	120 (40%)
Amasha et al. 2012 [18]	To determine the prevalence of the use of home remedies to relieve pregnancy-related complaints among pregnant Jordanian women.	Jordan	CSS; using a semi-structured questionnaire, women attending antenatal clinic	332 (NR)	198 (59.6%)
Hashem Dabaghian et al. 2012 [36]	To determine the prevalence of herbal medicine use in pregnant women attending some Tehran (Iran) governmental hospitals for prenatal care.	Iran	CSS; using a semi-structured questionnaire, women attending perinatal clinic	600 (27.03 ± 4.8)	402 (67%)
Hwang et al. 2016 [38]	To gain insights into the prevalence and factors leading to the use of complementary and alternative medicine (CAM) among pregnant women in Iraq.	Iraq	CSS; using structured questionnaire, women attending antenatal clinic	335 (26.1 ± 6.9)	180 (53.7%)
Orief et al. 2014 [49]	To elucidate the use of herbal medicines in pregnant women and to explore patterns of herbal medication use including dietary supplements in pregnant women in Alexandria, Egypt.	Egypt	CSS; using questionnaire, women attending family health center	300 (26.9 ± 4.9)	82 (27.3%)
Rahman et al. 2009 [19]	To determine whether the use of herbal medicines during pregnancy is associated with women’s attitude towards herbal medicines, and what are their sociodemographic features.	Malaysia	CSS; using structured questionnaire, women registered with birth registration record were surveyed during child health clinic sessions	210 (31 ± 6.5)	110 (52.4%)
Tabatabaee 2011 [42]	To evaluate the drug utilization pattern during pregnancy in Kazeroon, south of Iran.	Iran	CSS; using structured questionnaire, two days after childbirth at postnatal ward	513 (25.7 ± 4.7)	158 (30.8%)
Total				2729	1283 (47.01%)

CSS = Cross-sectional survey; NR = Not reported

Mean age reported in years (mean ± standard deviation)

### Most frequently used herbal medicines

In total, 1283 out of 2729 (47.01%) women used at least one herbal medicine any time during their last pregnancy. A long list of herbal medicines was identified from the reviewed articles. In this review, we only report those modalities that were used by 10 or more subjects. As a result, 31 different herbal medicines (individual herb or mixture as preparation) were determined, which are presented in Table 4. Most frequently used herbal medicines included peppermint (292), aniseed (188), olibanum (166) and flixweed seed (156). Nearly all of the herbal medicines were administered via oral route, whereas only two were used topically, one as an inhalation and one through vaginal route.

**Table 4:** The most frequently used herbs, route of administration and reported traditional indications during pregnancy

No.	Herb^a^	Number of users (Total = 1283)	Route	Time of use (Trimester of gestation)^b^	Reported traditional use
1	Peppermint (*Mentha piperita*)^2, 3, 4, 5, 6, 8^	292 (22.8%)	Oral	1st, 2nd, 3rd	Flu, cough, heartburn, bloating, flatulence, stomach/abdominal pain, nausea, vomiting, facilitate delivery, relaxation
2	Aniseed (*Pimpinella anisum*)^1, 2, 3, 6^	188 (14.7%)	Oral	1st, 2nd, 3rd	Flu, cough, stomach/abdominal pain, vomiting, diuretic, chest pain, laxative, flatulence, infections, relaxation
3	Olibanum (Frankincense - *Boswellia sacra*)^4, 8^	166 (12.9%)	Oral	1st, 2nd	Increasing neonate’s intelligence
4	Flixweed seed (*Descurainia sophia*)^4, 8^	156 (12.2%)	Oral	3rd	Cold, constipation, prevention of neonatal hyperbilirubinemia
5	Ginger (*Zingiber officinale*)^1,2,3,4,5,6,8^	147 (11.5%)	Oral	1st, 2nd, 3rd	Flu, cold, cough; nausea, vomiting; weight reduction
6	Chamomile (*Matricaria chamomilla*)^2, 5, 8^	121 (9.4%)	Oral	1st, 3rd	Flu, cough, stomach/abdominal pain, vomiting, diuretic, chest pain, laxative, flatulence, pharyngitis, relaxation
7	Sage (*Salvia officinalis*)^2, 3^	112 (8.7%)	Oral, vaginal	NR	Flu, vomiting, heartburn, abdominal pain, infections, teeth pain
8	Cinnamon (*Cinnamomum verum*)^2, 4, 5, 8^	100 (7.8%)	Oral	1st, 2nd, 3rd	Anemia, bloating, stomach/abdominal pain, laxative, facilitate delivery
9	Fenugreek (*Trigonella foenum-graecum*) ^2, 3, 6^	80 (6.2%)	Oral	1st, 2nd, 3rd	Cough, infections, constipation, piles,
10	Black seed (*Nigella sativa*)^1, 3, 5^	79 (6.2%)	Oral	NR	Colic, gases, nutritional supplement, infections
11	Pennyroyal (*Mentha pulegium*)^4^	78 (6.1%)	Oral	1st, 2nd, 3rd	Breathing problems
12	Coconut oil (*Cocos nucifera*)^7^	69 (5.4%)	Oral, topical	NR	Nausea, vomiting, heartburn, constipation, smooth body and hair
13	Borage (*Borago officinalis*)^4, 8^	66 (5.1%)	Oral	1st, 2nd, 3rd	Cold, constipation, tranquilizer
14	Thyme (*Thymus vulgaris*)^1, 2, 3, 5^	56 (4.4%)	Oral	NR	Flu, cough, digestive disorders, infections (pharyngitis, urinary tract, bronchitis)
15	Ammi (*Ammi visnaga*)^8^	50 (3.9%)	Oral	1st, 2nd, 3rd	Nausea, vomiting, and other gastrointestinal problems
16	Chicory (*Cichorium intybus*)^4^	47 (3.7%)	Oral	3rd	Prevention of neonatal jaundice
17	Green tea (*Camellia sinensis*)^4, 6^	47 (3.7%)	Oral	1st, 2nd, 3rd	Sedative
18	Chahar tokhmeh [Quince + Alyssum + Greater plantain + Basil] *(Cydonia oblonga + Lobularia maritima + Plantago major + Ocimum basilicum)* ^4^	41 (3.2%)	Oral	1st, 2nd, 3rd	Respiratory infections
19	Garlic (*Allium sativum*)^5, 6^	37 (2.9%)	Oral	1st, 2nd, 3rd	Enhance immune system for herself and healthy baby
20	Dates (*Phoenix dactylifera*)^2^	34 (2.6%)	Oral	NR	Energy, facilitate delivery, laxative
21	Castor oil (*Ricinus communis*)^5^	33 (2.6%)	Oral	NR	Induce labor
22	Egyptian willow (*Salix aegyptiaca*)^4^	32 (2.5%)	Oral	3rd	Sedative
23	Licorice (*Glycyrrhiza glabra*)^4, 5, 8^	31 (2.4%)	Oral	1st, 2nd, 3rd	Cold, bloating, stomach-ache
24	Basil *(Ocimum basilicum)* ^8^	28 (2.2%)	Oral	2nd, 3rd	Prevention of neonatal hyperbilirubinemia
25	Oregano (*Origanum vulgare*)^4^	27 (2.1%)	Oral	1st, 2nd, 3rd	Cough
26	Cumin (*Cuminum cyminum*)^2, 5, 8^	24 (1.9%)	Oral	1st, 2nd	Flatulence, abdominal pain, facilitate labor
27	Jujube (*Zyzyphus jujube*)^4^	23 (1.8%)	Oral	1st	Nausea
28	Aloe (*Aloe vera*)^4^	18 (1.4%)	Topical	3rd	Skin cracks
29	Kacip Fatimah (*Labisia pumila*)^7^	13 (1.0%)	Oral	NR	Facilitate labor, loss of libido
30	Eucalyptus (*Eucalyptus globulus*)^4^	12 (0.9%)	Inhalation	1st, 2nd, 3rd	Breathing problems
31	Olive oil (*Olea europaea*)^5^	11 (0.9%)	Oral	NR	Healthy development of fetus

^a^superscript numbers from 1 to 8 on every herbal modality indicate the study which reported use of that modality: Al-Riyami et al.^1^ *;* Jaradat et al.^2^ *;* Amasha et al.^3^ *;* Hashem Dabaghian et al.^4^ *;* Hwang et al.^5^ *;* Orief et al.^6^; Rahman et al.^7^ *;* Tabatabaee^8^

^b^please note that information on time of use of the herbs was available only from three studies [36, 42, 49]

NR = Not reported

### Indications of using herbal medicines

The studies reported a broad range of indications for using herbal medicines by women during pregnancy. The most common indications include nausea/vomiting, abdominal pain, preventing neonatal hyperbilirubinemia, breathing problems, cold/flu/cough, bloating/flatulence, as a relaxant, to facilitate labour and to enhance neonate’s intelligence. Reported traditional indications of most frequently used herbal medicines are presented in Table 4.

### Safety classification

**Fig. 2:**
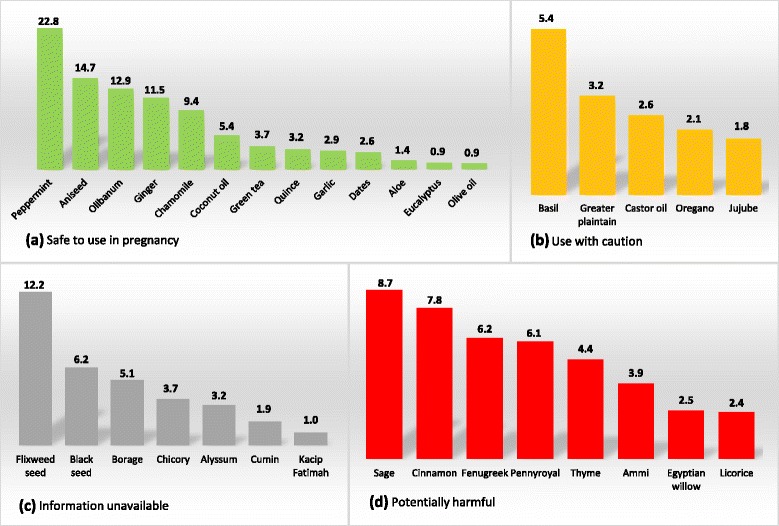
Frequency of herbal medicines used in pregnancy according to their safety classification. **a** Herbal medicines in this section are classified as safe to use in pregnancy. **b** Herbal medicines in this section should not be used without consulting a qualified health care practitioner. **c** Information on safety for herbal medicines in this section is not available in current literature. **d** Herbal medicines in this section can be harmful to mother or fetus. Note: Number of herbal medicine users for each type is given in percentage out of total number of users

Details on safety classification of most frequently used herbal medicines during pregnancy are given in Table 5. Out of 33 individual herbs, only 13 were classified as safe to use in pregnancy. For seven herbal medicines that included flixweed seed, black seed, chicory and others, there was insufficient information in current literature. Frequency of the herbal medicines that were classified according to each safety category is presented in Fig. 2. In total, eight herbal medicines were classified as potentially harmful to use in pregnancy. These included pennyroyal, licorice, sage, ammi and others. Out of these eight herbal medicines, human studies reporting harmful effects were available for only two herbs; the rest were categorized based on animal studies or their potential to cause harm to mother and/or fetus.

**Table 5:** Documentation on safety of most frequently used herbs during pregnancy

Sr.	Herb (or mixture)	Study subjects in references studies	Documentation on safety
Safe to use in pregnancy			
1	Ginger (*Zingiber officinale*)	Human	Clinical evidence in human pregnancy have not found any harmful effect to mother or fetus [28, 50, 51].
2	Garlic (*Allium sativum*)	Human	Studies in human pregnancy have shown no adverse effect of garlic [24, 52].
3	Dates (*Phoenix dactylifera*)	Human	One prospective human study did not report any harmful effect on mother and fetus [53].
4	Olive (*Olea europaea*)	Human	Clinical human evidence have not found any harmful effect to mother or fetus [54, 55].
5	Coconut oil (*Cocos nucifera*)	Human	No health hazards are reported in conjunction with the use of coconut oil as food or drug or even in raw form [28].
6	Aloe (*Aloe vera*)	Human	Topical application by pregnant women is unlikely to be harmful [29]. However, it should not be taken orally during pregnancy as the aloe latex contains anthraquinones that may stimulate uterus and initiate premature labor or possibly cause abortion [28].
7	Peppermint (*Mentha piperita*)	Human	Evidence in human pregnancy following use as tea has not shown any harmful effect to mother or fetus [16, 25–27]. Excessive dose should be avoided due to its emmenagogue properties [28].
8	Aniseed (*Pimpinella anisum*)	Human	Safe to use in human pregnancy with normal doses [16, 25, 26]. It increases the action of warfarin, so it is not recommended for women on warfarin [56].
9	Olibanum (Frankincense - *Boswellia sacra*)	Human	Not harmful to human mother or fetus in moderate doses for mild ailments [25]. Its resin in high doses is an emmenagogue and may induce abortion [29].
10	Chamomile (*Matricaria chamomilla*)	Human	Can be safely used as tea in moderate amounts during in human pregnancy [16, 25, 27]. It may act as a uterine stimulant, so large doses in pregnancy should be avoided [29]. Prolonged use has been related with premature constriction of fetal ductus arteriosus [57].
11	Quince (*Cydonia oblonga*)	Human	A recent controlled study has shown benefit against mild nausea and vomiting in human pregnancy without any adverse effect [58].
12	Green tea (*Camellia sinensis*)	Human	Safe to use as tea in moderate quantity [16]. Pregnant women are recommended to avoid large quantities due to the caffeine content [28].
13	Eucalyptus (*Eucalyptus globulus*)	Human	Should only be used topically [16, 25]. In rare cases, oral ingestion may cause nausea, vomiting and diarrhea [30]. Due to known toxicity and unknown effects during pregnancy, its ingestion should be avoided [29].
Use with caution			
1	Basil (*Ocimum basilicum)*	NA	It has not been studied in human pregnancy and should not be used in doses higher than commonly found in food [16, 25].
2	Greater plantain (*Plantago major*)	NA	The herb has not been studied in human pregnancy, although no harmful contents have been identified. Therefore, it can be used but with caution [16, 25].
3	Oregano (*Origanum vulgare*)	NA	It has not been studied in human pregnancy and should not be used in doses higher than commonly found in food [16, 25, 27].
4	Castor oil (*Ricinus communis*)	Human	Human studies have indicated use of castor oil to induce labor, however, it should not be used without proper supervision of a qualified health care practitioner [25]. Over dosage can lead to severe gastric irritation with vomiting, colic and severe diarrhea [28].
5	Jujube (*Zyzyphus jujube*)	NA	No scientific report available on its use and safety during pregnancy. Evidence regarding safety has not been conclusively established [25]. Therefore, it should be only used in pregnancy with supervision of a qualified health care practitioner.
Information unavailable about safety in pregnancy			
1	Flixweed seed (*Descurainia sophia*)	NA	No scientific report available on its use and safety during pregnancy.
2	Black seed (*Nigella sativa*)	NA	No scientific report available on its use and safety during pregnancy in humans. Traditionally believed to slow down or stop uterus from contracting if taken in doses higher than commonly found in food.
3	Kacip Fatimah (*Labisia pumila*)	NA	No scientific report available on its use and safety during pregnancy.
4	Cumin (*Cuminum cyminum*)	NA	Information regarding safety in human pregnancy is lacking. In India, it is used as an abortifacient [28]. Large doses in animal studies have shown antifertility activities [59]. Therefore, doses higher than commonly found in food should be avoided.
5	Chicory (*Cichorium intybus*)	NA	Not studied in human pregnancy, so the safety has not been conclusively established [25, 26].
6	Borage (*Borago officinalis*)	NA	Information regarding safety in human pregnancy is lacking [25]. Should be avoided during pregnancy due to possible teratogenic and labor inducing effects of prostaglandin E agonists [60].
7	Alyssum (*Lobularia maritima*)	NA	No scientific report available on its use and safety during pregnancy.
Potentially harmful in pregnancy			
1	Pennyroyal (*Mentha pulegium*)	Human	Use of the volatile oil in pregnancy is not recommended as it has been reported to cause abortion if taken in high doses; cases of death have been reported following misuse of its volatile oil to induce abortion [28]. It contains potentially toxic compound pulegone and should be avoided [25].
2	Licorice (*Glycyrrhiza glabra*)	Human	Not recommended during pregnancy because of possible alterations of hormone levels and the association with preterm delivery [27–30, 39].
3	Sage (*Salvia officinalis*)	Human	Not to be used during pregnancy due to abortifacient properties [16, 27, 28]. The pure essential oil and alcoholic extracts should not be taken during pregnancy [30].
4	Ammi (*Ammi visnaga*)	NA	Human or animal studies not available in current literature but its active constituent, khellin, has uterine stimulating activity; therefore, it is contraindicated during pregnancy [61].
5	Thyme (*Thymus vulgaris*)	NA	Human or animal studies not available in current literature but potentially harmful due to its abortifacient activity [16, 25, 26].
6	Fenugreek (*Trigonella foenum-graecum*)	Animal	Evidence suggests abortifacient effects as one animal study showed stimulating effects on uterus [25, 27]. It also possesses hypoglycemic, hypolipidemic and hypothyroid properties [16].
7	Cinnamon (*Cinnamomum verum*)	Animal	Animal evidence suggests possibility of fetal malformation following ingestion of its essential oil [16, 25]. Should only be used in doses commonly found in food.
8	Egyptian willow (*Salix aegyptiaca*)	NA	No human or animal study found for this particular herb. No safety data on use during pregnancy exists for its counterpart white willow. Nevertheless, consumption of both of these in pregnancy should be avoided as these contain salicylates which can cross the placenta [16, 25].

NA = Not available

## Discussion

Systematic reviews usually focus on discovering the effectiveness of interventions [34]. To the best of our understanding, this is the first systematic review to identify the most commonly used herbal medicines in pregnancy in the Asian countries and classify them according to their safety status based on reference sources. The review comprised of eight cross-sectional studies that included data on 2729 women. The findings suggest that use of herbal medicines during pregnancy can have a number of adverse effects to mother and/or fetus.

Out of 2729, 1283 (47.01%) women used at least one herbal medicine during their previous pregnancy. This study classified the safety status of 33 most common herbal medicines used in pregnancy. Variety of herbal medicines exist among different countries due to various factors such as historical beliefs, local traditions, and religion [35, 36]. For instance, use and cultivation of black seed is mentioned in the *Qur’an* and the *Bible*, and it has been believed to possess medicinal properties such as antidiabetic, antimicrobial and gastro protective effects [37]. In one of the studies included in this review, black seed was the most frequently used medicinal herb in pregnancy [38]. Out of 33 individual herbs, only 13 were classified as safe to use in pregnancy, whereas eight were potentially harmful.

Peppermint was the most frequently used herbal medicine. It was also the most popular among various countries as six out of eight studies reported its use. Pregnant women used peppermint to manage various common conditions such as flu, heartburn, stomach pain, nausea and vomiting. In this study, it was classified as safe to use in pregnancy as studies have not reported any harmful event following maternal consumption of peppermint or its tea [25]. However, its higher doses should be avoided. It is reported that if high doses of peppermint is ingested during pregnancy, it could trigger menstruation and cause abortion [28]. Aniseed was the second most frequently used modality, and like peppermint, it was consumed to manage various mild ailments. Literature suggests that within normal doses it can be safely used in pregnancy. It is believed that aniseed possesses estrogen like activity, but there is no scientific evidence to prove it. In the case of *Aloe Vera*, it should be noted that its ingestion is contraindicated during pregnancy [28]. The herb is harmless only if it is used topically [29]. Despite the evidence supporting safety of ‘safe to use’ herbal medicines, it is recommended that these should be taken only in normal doses [16].

Pennyroyal and licorice were also among the most frequently used herbal medicines and were classified as potentially harmful in pregnancy. These were consumed by pregnant women to manage breathing and digestion related problems respectively. In literature, use of pennyroyal is contraindicated during pregnancy [16] as cases of death have been reported following the misuse of its volatile oil to induce abortion [28]. Similarly, other studies have shown that ingestion of licorice during pregnancy can alter hormone levels and can lead to preterm delivery [39]. There is no scientific report to support the effectiveness of these herbal medicines especially among pregnant women.

In classifying the safety of herbal medicines, information on seven herbal medicines in the current literature was not sufficient. To be on the safe side, it is recommended that either use of these medicines be totally avoided during pregnancy or some herbs that are part of the everyday food such as black seed and cumin should be consumed only in doses commonly found in food. Moreover, five herbal medicines were classified in the ‘use with caution’ category. Available human evidence for these herbal medicines was limited so it is recommended that these should not be used without supervision of a qualified health care practitioner.

This systematic review has some limitations. The quality assessment tool which was used to appraise the quality of studies included, has been used in systematic reviews of observational studies previously [31, 32]. The tool is a modified version of Newcastle-Ottawa Scale for cross-sectional studies. The Newcastle-Ottawa Scale in its original form only determines basic quality indicators of the reviewed studies [40]. Whereas the modified version was considered more suitable to the purpose of this study, especially given the discrete nature of studies on herbal medicines. Moreover, it is an easily applicable and understandable instrument to appraise important methodological aspects of an observational study. However, only five of its items are standard whereas the rest of items are modifiable according to objective of the review. This can affect the scoring system and the scores may not be comparable across different systematic reviews.

Considerable discrepancies among the reviewed studies were found, for instance in terms of sample size, research design and survey instrument. The study from Oman recruited sample as low as 139 participants whereas one study from Iran recruited 600 participants [36, 41]. Four of the eight included studies surveyed women during antenatal period while rest of the studies recruited postnatal women. Among these, the study from Malaysia registered postnatal women using national birth register [19], whereas other studies using postnatal sample recruited participants from inpatient departments [17, 36, 42]. Malaysian study did not report the number of users of every herbal modality [19]. However, it was obtained from another report published elsewhere [43]. Furthermore, only three of the eight studies reported time of use of individual herbal medicine in pregnancy. The discrepancies among the reviewed studies were obvious and should be considered while interpreting the results of this review.

This review was designed to include cross-sectional studies. By doing so, several herbal medicines used in developing Asian countries were identified and classified according to their safety status for use in pregnancy. Healthcare professionals and researchers can disseminate the results of this study and play a key role to create awareness on prevention of unwanted effects of herbal medicines, which is a strength of this study.

Considering the popularity of using herbal medicines and scarcity of scientific evidence supporting its efficacy and safety in pregnancy, it is very important to investigate their potential effects on mother and the neonate. An Italian study reported neonatal characteristics, malformations, complications during birth, difference in morbidities between users of herbal medicine and non-users and potential side effects of using herbal medicines [12]. In addition to that, Holst et al. reported possible impact of using herbal medicines in early pregnancy on the pregnancy outcome [44]. However, among the studies included in this review, only one study investigated basic indicators such as gestational age at birth, mode of delivery, and weight of newborn [17]. Although the rest of the reviewed studies and most of the other cross-sectional studies employed sample of postpartum women, none investigated outcome and potential adverse effects of herbs on mother and fetus [19, 21, 36, 42, 45, 46].

The trend of not reporting effect of herbal medicine could be due to the cultural bias. Studies on traditional medicine are usually conducted by researchers coming from that region. The affinity to that region may affect their judgement of the benefits and risks involved [47]. Thus focusing on popularity of the tradition and ignoring its potential risks. Moreover, institutions and researches are regularly evaluated based on the number of publications. So there is a growing pressure on researchers to frequently publish their research in high rank journals [48]. Consequently, authors may want to avoid reporting of less popular and relatively controversial findings that might provoke debate with the reviewers and could possibly result in rejection of the manuscript. This in turn may confine researchers to report only prevalence, knowledge, and attitudes regarding use of traditional medicine, which might be the case with studies included in this review. Authors may also restrict their focus to prevalence and attitude type of studies due to the lack of a standard methodological tool for evaluating the outcome and potential adverse effects of herb use during pregnancy. In this case, there is need to develop a standard tool such as a questionnaire instrument for measuring the outcome and potential effects of using herbal medicines during pregnancy. The authors of this study are working on another research to develop such standard survey instrument. The instrument will be pilot tested among culturally diverse populations of the developing Asian countries in the near future.

## Conclusions

Herbal medicines may be natural but do contain pharmacologically active ingredients. Several herbal medicines identified in this review were classified to be potentially harmful or the safety information in pregnancy was missing. It is recommended that herbal medicines that are classified as safe to use should be taken only in normal doses. Whereas contraindicated herbal medicines should be avoided and other herbals should be taken under supervision of a qualified health care practitioner. Healthcare professionals and researchers can disseminate the results of this study and play a key role to create awareness on prevention of unwanted effects of herbal medicines. Given the scarcity of studies, it is recommended that future studies should focus on effects of herbal medicines on pregnancy outcome and their potential harmful effects.

## Additional files

Additional file 1: PRISMA checklist (DOC 63 kb)

Additional file 2: Table S1. PubMed/MEDLINE search strategy for studies to include in systematic review. **Table S2.** PubMed/MEDLINE search strategy to evaluate the safety status of identified herbs (DOCX 18 kb)

## Abbreviations

CSS: Cross-sectional survey
NR: Not reported
PRISMA: Preferred Reporting Items for Systematic Reviews and Meta-Analyses
RCT: Randomized controlled trials

## References

[1] Ben-Arye E, Lev E, Schiff E (2011). Complementary medicine oncology research in the middle-east: shifting from traditional to integrative cancer care. European Journal of Integrative Medicine.

[2] Kuo GM, Hawley ST, Weiss LT, Balkrishnan R, Volk RJ (2004). Factors associated with herbal use among urban multiethnic primary care patients: a cross-sectional survey. BMC Complement Altern Med.

[3] AlBraik FA, Rutter PM, Brown D (2008). A cross-sectional survey of herbal remedy taking by united Arab emirate (UAE) citizens in Abu Dhabi. Pharmacoepidemiol Drug Saf.

[4] Cuzzolin L, Benoni G: Safety issues of phytomedicines in pregnancy and paediatrics. In: Herbal drugs: ethnomedicine to modern medicine. Edn.: springer; 2009: 381–396.

[5] Bogusz MJ, Al Tufail M, Hassan H (2002). How natural are ‘natural herbal remedies’?. Adverse Drug React Toxicol Rev.

[6] Hall HG, Griffiths DL, McKenna LG (2011). The use of complementary and alternative medicine by pregnant women: a literature review. Midwifery.

[7] Ernst E (2002). Herbal medicinal products during pregnancy: are they safe?. BJOG.

[8] Choudhry UK (1997). Traditional practices of women from India: pregnancy, childbirth, and newborn care. J Obstet Gynecol Neonatal Nurs.

[9] Marcus DM, Snodgrass WR (2005). Do no harm: avoidance of herbal medicines during pregnancy. Obstet Gynecol.

[10] Posadzki P, Watson LK, Ernst E (2013). Adverse effects of herbal medicines: an overview of systematic reviews. Clin Med (Northfield Il).

[11] Peters D (2009). CAM: doing more good than harm. Focus Altern Complement Ther.

[12] Cuzzolin L, Francini-Pesenti F, Verlato G, Joppi M, Baldelli P, Benoni G (2010). Use of herbal products among 392 Italian pregnant women: focus on pregnancy outcome. Pharmacoepidemiol Drug Saf.

[13] Mabina M, Pitsoe S, Moodley J (1997). The effect of traditional herbal medicines on pregnancy outcome. The king Edward VIII hospital experience. South African medical journal= Suid-Afrikaanse tydskrif vir geneeskunde.

[14] Finkel RS, Zarlengo KM (2004). Blue cohosh and perinatal stroke. N Engl J Med.

[15] Nordeng H, Havnen GC (2004). Use of herbal drugs in pregnancy: a survey among 400 Norwegian women. Pharmacoepidemiol Drug Saf.

[16] Kennedy D, Lupattelli A, Koren G, Nordeng H (2016). Safety classification of herbal medicines used in pregnancy in a multinational study. BMC Complement Altern Med.

[17] Jaradat N, Adawi D (2013). Use of herbal medicines during pregnancy in a group of Palestinian women. J Ethnopharmacol.

[18] AMASHA H, JARRAH S. The use of home remedies by pregnant mothers as a treatment of pregnancy related complaints: an exploratory study. The Medical Journal of Cairo University. 2012;80(2)

[19] Rahman AA, Sulaiman SA, Ahmad Z, Salleh H (2009). Daud WNW.

[20] Moher D, Liberati A, Tetzlaff J, Altman DG, Group P (2009). Preferred reporting items for systematic reviews and meta-analyses: the PRISMA statement. PLoS Med.

[21] Louik C, Gardiner P, Kelley K, Mitchell AA: Use of herbal treatments in pregnancy. Am J Obstet Gynecol 2010, 202(5):439. e431–439. e410. 10.1016/j.ajog.2010.01.055 PMC2867842 20452484

[22] Forster DA, Denning A, Wills G, Bolger M, McCarthy E (2006). Herbal medicine use during pregnancy in a group of Australian women. BMC Pregnancy Childbirth.

[23] Kennedy DA, Lupattelli A, Koren G, Nordeng H (2013). Herbal medicine use in pregnancy: results of a multinational study. BMC Complement Altern Med.

[24] Mills E, Dugoua J-J, Perri D, Koren G: Herbal medicines in pregnancy and lactation: an evidence-based approach: CRC press; 2013.

[25] Gardner Z, McGuffin M: American herbal products Association’s botanical safety handbook: CRC press; 2013.

[26] European Medicines Agency [http://www.ema.europa.eu/ema/]. Last accessed: 13 Aug 2017.

[27] Natural medicine In: Natural Medicine. Somerville, MA: Natural Medicine https://naturalmedicines.therapeuticresearch.com/databases/food,-herbs-supplements/. Last accessed: 11 Aug 2017.

[28] Gruenwald J, Brendler T, Jaenicke C: PDR for herbal medicines: Thomson, Reuters; 2007.

[29] Ulbricht CE, Basch EM: Natural standard herb & supplement reference: evidence-based clinical reviews: Mosby; 2005.

[30] Wichtl M: Herbal drugs and phytopharmaceuticals: a handbook for practice on a scientific basis: CRC press; 2004.

[31] Saab MM, Landers M, Hegarty J: Testicular cancer awareness and screening practices: a systematic review. In: Oncol Nurs Forum*:* 2016; 2016. 10.1188/16.ONF.E8-E23 26679456

[32] Davids EL, Roman NV (2014). A systematic review of the relationship between parenting styles and children's physical activity. African Journal for Physical Health Education. Recreation and Dance.

[33] Traditional medicine**:** Definitions [http://www.who.int/medicines/areas/traditional/definitions/en/]**.** Last accessed**:** 06 Aug 2017**.**

[34] Loke YK, Price D, Herxheimer A (2007). Systematic reviews of adverse effects: framework for a structured approach. BMC Med Res Methodol.

[35] Sharma V, Joshi B (2010). Role of sacred plants in religion and health care system of local people of Almora district of Uttarakhand state (India). Academic Arena.

[36] Hashem Dabaghian F, Abdollahi Fard M, Shojaei A, Kianbakht S, Zafarghandi N, Goushegir A (2012). Use and attitude on herbal medicine in a group of pregnant women in Tehran. Journal of Medicinal Plants.

[37] Yarnell E, Abascal K (2011). Nigella Sativa: holy herb of the middle east. Alternative and Complementary Therapies.

[38] Hwang JH, Kim Y-R, Ahmed M, Choi S, Al-Hammadi NQ, Widad NM, Han D (2016). Use of complementary and alternative medicine in pregnancy: a cross-sectional survey on Iraqi women. BMC Complement Altern Med.

[39] Strandberg TE, Andersson S, Järvenpää A-L, McKeigue PM (2002). Preterm birth and licorice consumption during pregnancy. Am J Epidemiol.

[40] Wells G, Shea B, O’connell D, Peterson J, Welch V, Losos M, Tugwell P: The Newcastle-Ottawa Scale (NOS) for assessing the quality if nonrandomized studies in meta-analyses. 2009. Epub Available from: URL: http://www ohri ca/programs/clinical_epidemiology/oxford htm [cited 2009 Oct 19] 2013.

[41] Al-Riyami IM, Al-Busaidy IQ, Al-Zakwani IS (2011). Medication use during pregnancy in Omani women. Int J Clin Pharm.

[42] Tabatabaee M (2011). Use of herbal medicine among pregnant women referring to Valiasr hospital in Kazeroon, Fars, south of Iran. Journal of Medicinal Plants.

[43] Ab Rahman A, Ahmad Z, Naing L, Sulaiman SA (2007). Hamid AM.

[44] Holst L, Nordeng H, Haavik S (2008). Use of herbal drugs during early pregnancy in relation to maternal characteristics and pregnancy outcome. Pharmacoepidemiol Drug Saf.

[45] Mothupi MC (2014). Use of herbal medicine during pregnancy among women with access to public healthcare in Nairobi, Kenya: a cross-sectional survey. BMC Complement Altern Med.

[46] Nordeng H, Havnen GC (2005). Impact of socio-demographic factors, knowledge and attitude on the use of herbal drugs in pregnancy. Acta Obstet Gynecol Scand.

[47] Tilburt JC, Kaptchuk TJ (2008). Herbal medicine research and global health: an ethical analysis. Bull World Health Organ.

[48] Lawrence PA (2003). The politics of publication. Nature.

[49] Orief YI, Farghaly NF, Ibrahim MIA (2014). Use of herbal medicines among pregnant women attending family health centers in Alexandria. Middle East Fertility Society Journal.

[50] Heitmann K, Nordeng H, Holst L (2013). Safety of ginger use in pregnancy: results from a large population-based cohort study. Eur J Clin Pharmacol.

[51] Viljoen E, Visser J, Koen N, Musekiwa A (2014). A systematic review and meta-analysis of the effect and safety of ginger in the treatment of pregnancy-associated nausea and vomiting. Nutr J.

[52] Aalami-Harandi R, Karamali M, Asemi Z (2015). The favorable effects of garlic intake on metabolic profiles, hs-CRP, biomarkers of oxidative stress and pregnancy outcomes in pregnant women at risk for pre-eclampsia: randomized, double-blind, placebo-controlled trial. J Matern Fetal Neonatal Med.

[53] Al-Kuran O, Al-Mehaisen L, Bawadi H, Beitawi S, Amarin Z (2011). The effect of late pregnancy consumption of date fruit on labour and delivery. J Obstet Gynaecol.

[54] Olsen SF, Østerdal ML, Salvig JD, Mortensen LM, Rytter D, Secher NJ, Henriksen TB (2008). Fish oil intake compared with olive oil intake in late pregnancy and asthma in the offspring: 16 y of registry-based follow-up from a randomized controlled trial. Am J Clin Nutr.

[55] Taavoni S, Soltanipour F, Haghani H, Ansarian H, Kheirkhah M (2011). Effects of olive oil on striae gravidarum in the second trimester of pregnancy. Complement Ther Clin Pract.

[56] Skidmore-Roth L: Mosby's handbook of herbs & natural supplements: Elsevier Health Sciences; 2009.

[57] Sridharan S, Archer N, Manning N (2009). Premature constriction of the fetal ductus arteriosus following the maternal consumption of camomile herbal tea. Ultrasound Obstet Gynecol.

[58] Jafari-Dehkordi E, Hashem-Dabaghian F, Aliasl F, Aliasl J, Taghavi-Shirazi M, Sadeghpour O, Sohrabvand F, Minaei B, Ghods R. Comparison of quince with vitamin B6 for treatment of nausea and vomiting in pregnancy: a randomised clinical trial. J Obstet Gynaecol. 2017;37(8):1048–52. 10.1080/01443615.2017.1322046 28631509

[59] Al Khamis K, Al Said M, Islam M, Tariq M, Parmar N, Ageel A (1988). Antifertility, antimplantation and abortifacient activity of the aqueous extracts of Cuminum Cyminum. Fitoterapia.

[60] Kast RE (2001). Borage oil reduction of rheumatoid arthritis activity may be mediated by increased cAMP that suppresses tumor necrosis factor-alpha. Int Immunopharmacol.

[61] Bhagavathula AS, Al-Khatib AJM, Elnour AA, Al Kalbani NM, Shehab A (2015). Ammi Visnaga in treatment of urolithiasis and hypertriglyceridemia. Pharm Res.

