# Supplementary material for: Safety classification of herbal medicines used among pregnant women in Asian countries: a systematic review
Source: BMC Complement Altern Med. 2017 Nov 14;17:489. doi: 10.1186/s12906-017-1995-6 (PMC5686907; doi:10.1186/s12906-017-1995-6)
Supplement: Supplementary file 2 — PubMed/MEDLINE search strategy for studies to include in systematic review. Table S2. PubMed/MEDLINE search strategy to evaluate the safety status of identified herbs (DOCX 18 kb) [file 12906_2017_1995_MOESM2_ESM.docx]

**Supplementary Data (Web Only Files)**

Mansoor Ahmed, Jung Hye Hwang, Soojeung Choi, Dongwoon Han. **Safety classification of herbal medicines used among pregnant women in Asian Countries: A Systematic Review**

**Table S1. PubMed/MEDLINE search strategy for studies to include in systematic review**

| 1. Pregnancy [MeSH Terms] 2. Pregnancy OR pregnant OR maternal OR reproductive 3. Herbal medicine [MeSH Terms] 4. Herbal medicine OR herbal preparations OR herbal tea OR medicinal herb OR medicinal plant OR phytomedicine 5. (names of developing Asian countries using OR in between) 6. Survey[pt] OR cross sectional survey[pt] OR cross-sectional survey[tiab]   (#1 OR #2) AND (#3 OR #4) AND #5 AND #6 |
| --- |

**Table S2. PubMed/MEDLINE search strategy to evaluate the safety status of identified herbs**

| 1. Pregnancy [MeSH Terms] 2. Pregnancy OR pregnant OR maternal OR reproductive 3. Herbal medicine [MeSH Terms] 4. (Herbal common name) 5. (Herbal Latin name)   (#1 OR #2) AND (#3) AND (#4 OR #5) |
| --- |
